# Recent progress in the design of photocatalytic H_2_O_2_ synthesis system

**DOI:** 10.3389/fchem.2022.1098209

**Published:** 2022-12-22

**Authors:** Haobing Wen, Sen Huang, Xianguang Meng, Xiaole Xian, Jingjing Zhao, Vellaisamy A. L. Roy

**Affiliations:** ^1^ Hebei Provincial Laboratory of Inorganic Nonmetallic Materials, College of Materials Science and Engineering, North China University of Science and Technology, Tangshan, China; ^2^ Traditional Chinese Medical College, North China University of Science and Technology, Tangshan, China; ^3^ School of Pharmacy, North China University of Science and Technology, Tangshan, China; ^4^ James Watt School of Engineering, University of Glasgow, Glasgow, United Kingdom

**Keywords:** photocatalysis, oxygen reduction reaction, H_2_O_2_ synthesis, cocatalyst, surface modification, ion doping

## Abstract

Photocatalytic synthesis of hydrogen peroxide under mild reaction conditions is a promising technology. This article will review the recent research progress in the design of photocatalytic H_2_O_2_ synthesis systems. A comprehensive discussion of the strategies that could solve two essential issues related to H_2_O_2_ synthesis. That is, how to improve the reaction kinetics of H_2_O_2_ formation *via* 2e^−^ oxygen reduction reaction and inhibit the H_2_O_2_ decomposition through a variety of surface functionalization methods. The photocatalyst design and the reaction mechanism will be especially stressed in this work which will be concluded with an outlook to show the possible ways for synthesizing high-concentration H_2_O_2_ solution in the future.

## 1 Introduction

**FIGURE 1:**
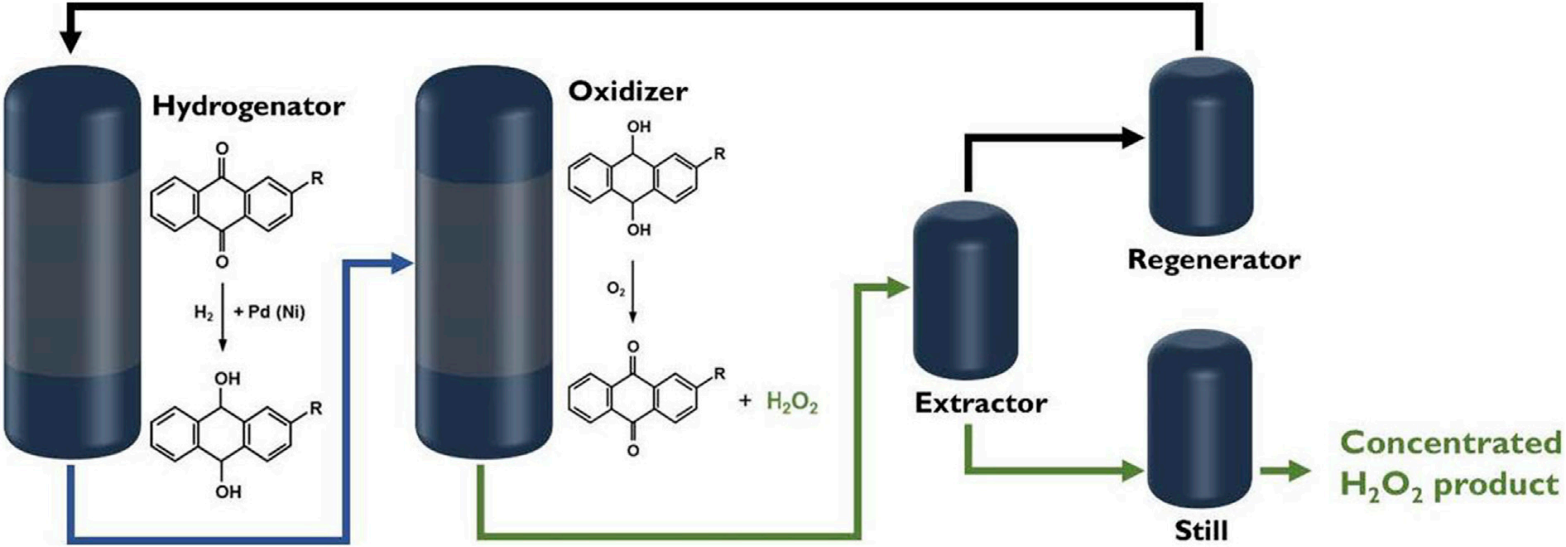
Flow chart of synthesis of H_2_O_2_ by AQ method (Yang et al., 2018).

H_2_O_2_ is an indispensable chemical in daily life. It has many applications in fields such as biology (Chang et al., 2021; Noh et al., 2020; Guarino et al., 2019), medicine (Andersen et al., 2006; Kozlova et al., 2015; Wang Y. et al., 2020), chemical industry (Chung et al., 2020; Zhang et al., 2021), environmental protection (Dinakar et al., 2020; Moreno, 2011). As a clean oxidant, the decomposition of H_2_O_2_ only yields H_2_O, which does not pose an environmental risk. Currently, the anthraquinone (AQ) method is the main method for the industrial production of H_2_O_2_ (Sterenchuk et al., 2018). The AQ method for H_2_O_2_ synthesis includes two steps: hydrogenation and oxidation (Campos-Martin et al., 2006; Halder and Lawal, 2007; Gao et al., 2020). In this method, AQ is used as an intermediate, and the hydrogenation reaction is first performed with palladium catalyst (Chen, 2008; Edwards and Hutchings, 2008; Han et al., 2015). Then, oxygen is added to oxidize the hydroanthraquinone (AQH_2_) back to AQ and produce H_2_O_2_ (Figure 1). However, the AQ method not only has the risk of explosion, but consumes a lot of energy and organic solvent (Chen, 2008; Jia et al., 2018). Therefore, it is crucial to develop a safe and direct method to synthesize H_2_O_2_. The methods of direct H_2_O_2_ synthesis mainly includes electrocatalysis (Apaydin et al., 2018; Du et al., 2020; Sun et al., 2020; Shu et al., 2021), photocatalysis (Chen et al., 2018; Kormann et al.), and thermal catalysis (Adams et al., 2021). The electrocatalytic H_2_O_2_ synthesis has a high yield but it needs to consume useful electricity. Thermocatalytic H_2_O_2_ synthesis from oxygen and hydrogen also faces the risk of explosion when mixing the gases. The emerging photocatalytic H_2_O_2_ synthesis only uses solar energy to drive reaction without introducing hydrogen.

During photocatalytic H_2_O_2_ synthesis, the electron is first excited from the valence band to the conduction band of the photocatalyst. Then, it participates in oxygen reduction reactions (ORR) on the surface to generate H_2_O_2_. H_2_O_2_ synthesis *via* oxygen reduction can undergo two pathways. The step-by-step single-electron pathway is the first one (Eqs. 1–3), which is characterized by the presence of superoxide (HO_2_•) intermediate. The other is the direct two-electron (2e^–^) pathway (Eq. 1 and Eq. 4). Which one of these occurs can be confirmed by detecting the intermediate HO_2_• (Viswanathan et al., 2012; Baran et al., 2018; Fukuzumi et al., 2018; Haider et al., 2019; Anantharaj et al., 2021; Yang, 2021; Guo et al., 2022).

$$\mathrm{Photocatalyst}+\mathrm{hv}\to \;e^{-}+h^{+}$$ (1)

$$O_{2\;}+e^{-}+H^{+}\to \;•OOH$$ (2)

$$•OOH+e^{-}+H^{+}\to \;H_{2}O_{2}$$ (3)

$$O_{2\;}+2e^{-}+2H^{+}\to \;H_{2}O_{2}$$ (4)

Photocatalytic H_2_O_2_ generation is also accompanied by a decomposition reaction, which is the root cause of poor reaction stability. The decomposition process includes photolysis and light-independent decomposition. Taking TiO_2_ as an example, photolysis can occur mainly in four ways (Ⅰ) photogenerated electrons reduce H_2_O_2_ to OH^−^and •OH; (Ⅱ) photogenerated holes oxidize hydrogen peroxide to O_2_ or superoxide radical •O_2_^−^; (Ⅲ) The titanium peroxide complex (Ti-OOH) formed on the surface by the interaction of TiO_2_ and H_2_O_2_ gradually degrades under visible light; (Ⅳ) direct decomposition of H_2_O_2_ under ultraviolet light. H_2_O_2_ can also be decomposed in ways independent of light, such as pH and temperature.

The formation and decomposition performance of hydrogen peroxide are closely related to the surface properties of semiconductor photocatalysts. First, the high selectivity of cocatalysts to 2e^−^ ORR is needed to improve the photocatalytic H_2_O_2_ formation. Second, the functional modifier on photocatalyst can inhibit the decomposition of H_2_O_2_. These strategies indicate that surface functionalization of photocatalysts is very important. Considering these issues, we review the recent advances in the design of photocatalysts for H_2_O_2_ synthesis in this work.

## 2 Effect of cocatalyst on photocatalytic activity

### 2.1 Noble metal cocatalysts

**FIGURE 2:**
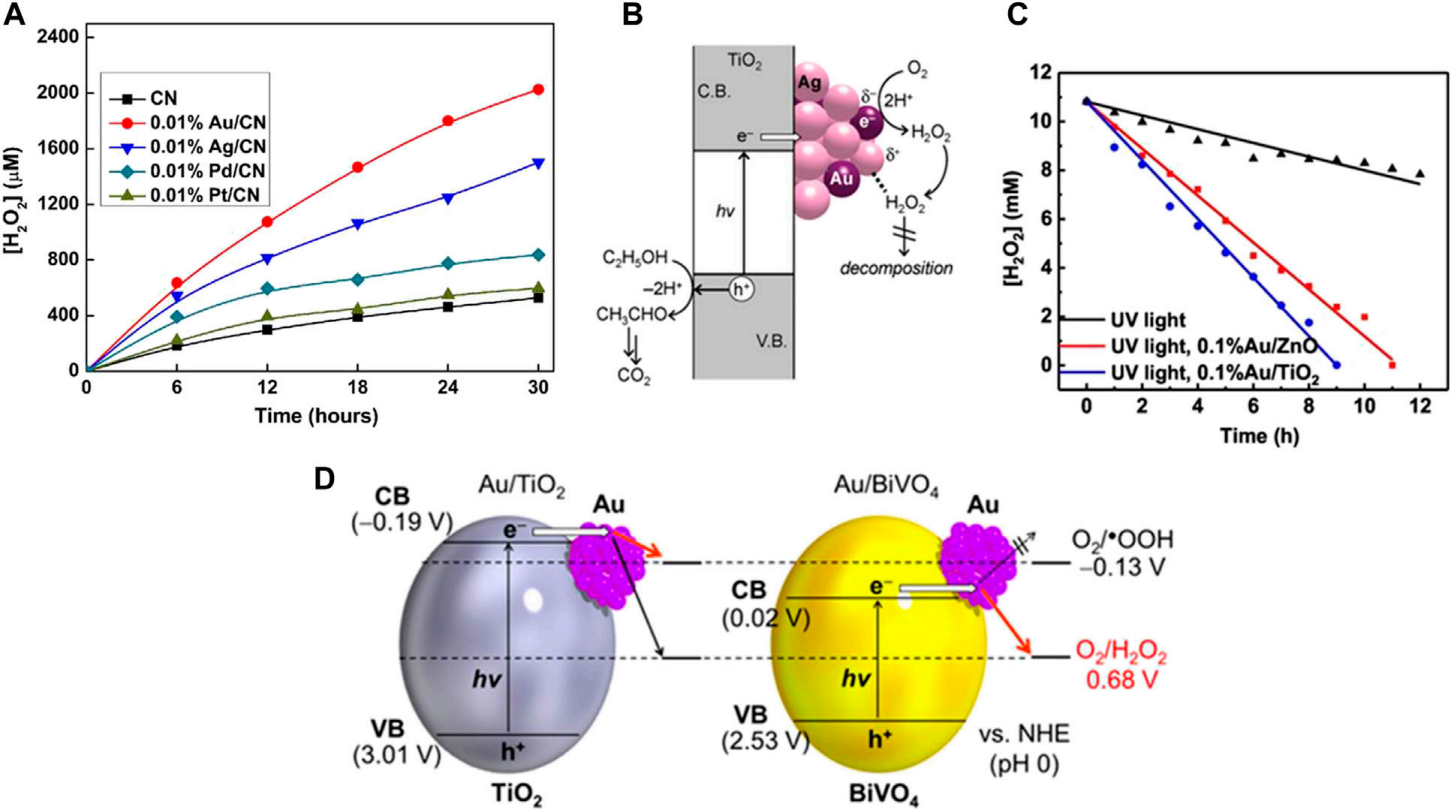
**(A)** Under visible light irradiation (λ > 420 nm), different noble metal loaded CN photocatalyzed H_2_O_2_ activity (Zuo et al., 2019a). **(B)** Mechanism for photocatalytic production of H_2_O_2_ on Au-Ag/TiO_2_ catalyst (Tsukamoto et al., 2012). **(C)** Photodecomposition of H_2_O_2_ under UV light with and without photocatalysts (Meng et al., 2020); **(D)** Energy diagrams for Au/TiO_2_ and Au/BiVO_4_, and reduction potential of O_2_ (Hirakawa et al., 2016).

Precious metals are widely used as cocatalysts in electrocatalysis and photocatalysis, while they also show excellent performance in ORR (Zinola et al., 1995; Chen et al., 2017a; Cai et al., 2019; Ignaczak et al., 2019; Jeon et al., 2020). Pt has good ORR performance and strong binding ability to intermediates such as O_2_ and OH•. When using Pt, generating H_2_O *via* 4e^–^ORR is favored, but it has poor selectivity for 2e^–^ORR (Kim J. et al., 2018; Chen J. Y. et al., 2020). Among these noble metals, Au has the best selectivity for 2e^−^ ORR, which has achieved efficient photocatalytic H_2_O_2_ synthesis in photocatalytic reaction (Jirkovsky et al., 2010; Jirkovsky et al., 2011; Zuo et al., 2019a; Ignaczak et al., 2019; Sun et al., 2020). Zuo et al. studied the influence of a series of noble metal co-catalysts (Pd, Pt, Au, and Ag) on the performance of photocatalytic H_2_O_2_ synthesis over g-C_3_N_4_. They found that the maximum activity could be achieved when the Au loading amount is very low (0.01 wt%) on g-C_3_N_4_ (Figure 2A) (Zuo et al., 2019a). A similar study showed that Au cocatalyst has the highest activity among different precious metals modified g-C_3_N_4_ samples (Kim H. I. et al., 2018). Similar high activity was observed over Au loaded TiO_2_-based photocatalysts (Tsukamoto et al., 2012), (Li L. et al., 2021; Feng et al., 2021).

The H_2_O_2_ yield of Au-Ag alloy cocatalyst supported on the surface of TiO_2_ was 2.3 times and 3.4 times higher than that of single Au or Ag cocatalyst. The reason was that the loaded Au-Ag alloy was conducive to the separation of electron holes, and the efficient photocatalytic reduction of O_2_ on Au atom promotes the formation of H_2_O_2_ (Figure 2B) (Tsukamoto et al., 2012). However, the activity of Au deposition on ZnO was better than that on TiO_2_, which is attributed to the more inert surface properties of ZnO than TiO_2_ when decomposing H_2_O_2_ (Figure 2C) (Meng et al., 2020).

Hirakawa et al. (Hirakawa et al., 2016) suggested that activity of Au cocatalyst is affected by the band structure of semiconductor photocatalytsts. They employed Au/BiVO_4_ photocatalyst to successfully produce H_2_O_2_ under visible light irradiation (λ> 420 nm). Since the conduction band potential of BiVO_4_ (0.02 V vs SHE) is more positive than the one-electron ORR potential (-0.13V) and more negative than the 2e^−^ ORR (0.68 V vs SHE), the 2e^−^ ORR can be selectively promoted while the one-electron ORR is inhibited. Compared with TiO_2_, BiVO_4_ has a narrower band gap, which indicates that BiVO_4_ has a better ability to utilize visible light and 2e ORR selectivity than TiO_2_ (Figure 2D).

### 2.2 Non-precious metal cocatalysts

Considering the scarcity and high cost of precious metals, developing non-precious metal co-catalysts for 2e^–^ORR is crucial (Zhang J. et al., 2020; Yan et al., 2020). For example, the surface of g-C_3_N_4_ was loaded with AQ as a cocatalyst. Its activity reached 361 μm/h, which was 4.4 times that of pure g-C_3_N_4_ and comparable to some precious metals. This is because, in addition to the 2e^−^ ORR reaction catalyzed by pure g-C_3_N_4_, another H_2_O_2_ synthesis pathway *via* hydrogenation (AQ + 2H^+^ + 2e^−^→AQH_2_) and dehydrogenation (AQH_2_ + O_2_→AQ + H_2_O_2_) plays a key role in the photocatalytic reaction (Kim H. I. et al., 2018). For CoP loaded on g-C_3_N_4_, the catalytic activity of CoP/g-C_3_N_4_ (70 μM•h^−1^) was similar to that of Au/g-C_3_N_4_ (67.56 μM•h^−1^) (Zuo et al., 2019a). This can be attributed to the accelerated separation and transfer of g-C_3_N_4_ photogenerated charge by CoP (Peng et al., 2017). The method of loading quantum dots to improve visible light absorption and electron mobility is also beneficial to photocatalytic synthesis of H_2_O_2_ (Zheng et al., 2018; Zhang M. M. et al., 2020; Liu et al., 2021a). Table 1 summarizes the effects of cocatalysts on hydrogen peroxide production activity.

**TABLE 1:** Activities of photocatalysts with different types of cocatalyst.

Photocatalyst	Catalyst mass	Incident light	Cocatalyst content	Reaction condition	H_2_O_2_ activity	Reaction mechanism^∗^	References
Ag@U-g-C_3_N_4_-NS-1.0	0.1 g (100 ml)	300 W Xe lamp (100 mW•cm^−2^)	Ag 1 wt%	pH = 3, O_2_, 1 mol/L HClO	1.975 × 10^–6^ M•min^−1^	—	Cai et al. (2019)
Au/C_3_N_4_-500(N_2_)	1 g/L	300 W Xe lamp (λ > 420 nm)	Au 2 wt%	pH = 3, O_2_, 5 vol% IPA	1320 μmol•L^−1^ (4 h)	—	Chang et al. (2018)
Au/Bi_2_O_3_-TiO_2_	200 mg (200 ml)	300 W Xe lamp (λ > 420 nm)	Au 0.1 wt%, Bi: Ti = 1 wt%	O_2_, 4 wt% C_2_H_5_OH	11 mM (12 h)	(Ⅱ)	Feng et al. (2021)
Au/BiVO_4_	50 mg (30 ml)	2 kW Xe lamp (λ > 420 nm)	Au 0.2 wt%	O_2_, 10 vol% EtOH	40.2 μM (10 h)	(Ⅰ)	Hirakawa et al. (2016)
Au/CN	400 mg (100 ml)	300 W Xe lamp (λ > 420 nm)	Au 0.01 wt%	pH = 8.5, O_2_, 10 vol% C_2_H_5_OH	2027 μM (30 h)	(Ⅰ)	Zuo et al. (2019a)
Au/β-CD-CN	0.4 g (100 ml)	2 kW Xe lamp (λ > 420 nm)	Au 0.05 wt%	O_2_, 10 vol% C_2_H_5_OH	3000 μM (30 h)	(Ⅰ)	Zuo et al. (2020)
Ag/β-CD-CN	0.4 g (100 ml)	300 W Xe lamp (λ > 420 nm)	Ag 0.05 wt%	O_2_, 10 vol% C_2_H_5_OH	1000 μM(30 h)	(Ⅰ)	Zuo et al. (2020)
Au/ZnO	0.2 g (200 ml)	300 W Xe lamp (UV-REF)	Au 0.1 wt%	O_2_, 4 wt% C_2_H_5_OH, 0.1M NaF	1.5 mmol^−1^•h^−1^	Au>0.1wt% (Ⅰ) Au<0.1wt% (Ⅱ)	Meng et al. (2020)
Au/SnO_2_-NR#TiO_2_	10 mg (10 ml)	300 W Xe lamp (λ > 430 nm)	—	O_2_, 4% EtOH	60 μM (6 h)	(Ⅰ)	Awa et al. (2020)
Au-Ag/TiO_2_	5 mg (5 ml)	450 W high pressure Hg lamp (λ > 280 nm)	Au 0.1 mol%, Ag 0.4 mol%	O_2_, 4 vol% C_2_H_5_OH	3.4 m′M (12 h)	(Ⅰ)	Tsukamoto et al. (2012)
Au@MoS_2_	0.05 g (50 ml)	300 W Xe lamp	Au 0.5 wt%	pH = 9, O_2_	1100 μM (12 h)	(Ⅰ) (Ⅲ)	Song et al. (2019)
Pt/TiO_2_	1 mg (20 ml)	500 W Hg lamp (λ > 300 nm)	Pt 1 wt%	Ar	5096 μmol•L^−1^•h^−1^	2H_2_O→H_2_+H_2_O_2_	Wang et al. (2019b)
Au-(ZT)-Al	5 cm × 5 cm	400–650 nm	17 wt%	pH = 7, 5 vol% C_2_H_5_OH	0.099 μM/min	—	Willis et al. (2020)
Pt-KCN(5)	0.2 g (200 ml)	400–800 nm	Pt 1 wt%	Remove air	620 μmol•g^−1^	(Ⅱ)	Hu et al. (2020)
Au/F-TiO_2_	0.2 g (200 ml)	300 W Xe lamp (λ > 420 nm)	Au 0.1wt%	O_2_, 4 wt% C_2_H_5_OH	6.5 mM (12 h)	(Ⅱ)	Li et al. (2021a)
Cu(hfacac)_2_/m-BiVO_4_	80 mg (80 ml)	300 W Xe lamp (λ > 430 nm)	400 μM Cu(hfacac)	O_2_, H_2_O:ACN:EtOH = 86:10:4	120 μM (2 h)	(Ⅰ)	Teranishi et al. (2020)
CoP/g-C_3_N_4_	20 mg (50 ml)	300 W Xe lamp (λ > 420 nm)	CoP 1.76 wt%	O_2_, 10 vol% C_2_H_5_OH	140 μM (2 h)	(Ⅱ)	Peng et al. (2017)
AQ/C_3_N_4_	0.5 g/L	100 mW•cm^−2^ 150 W Xe lamp	AQ 10 wt%	O_2_, 10 vol% IPA	361 μmol•L^−1^•h^−1^	(Ⅱ)	Kim et al. (2018b)
NiS@g-C_3_N_4_-30	10 mg (10 ml)	300 W Xe lamp (λ > 420 nm)	Ni 2.06 wt%	O_2_, 10 vol% C_2_H_5_OH	400 μM (1 h)	(Ⅱ)	Kim et al. (2018b)
Ti_3_C_2_/TiO_2_	50 mg (30 ml)	9 W white lamp (λ = 365 nm)	10% Ti_3_C_2_	O_2_, 10 vol% C_2_H_5_OH	359.43 μmol•h^−1^	(Ⅱ)	Chen et al. (2021)
SN-GQDs/TiO_2_	25 mg (50 ml)	500 W Xe lamp (λ > 300 nm)	SN-GQDs 0.5 wt%	pH = 3, O_2_, 6 vol% IPA	451 μM (60 min)	(Ⅱ)	Zheng et al. (2018)
FeOOH QDs/CQDs/g-C_3_N_4_	25 mg (100 ml)	300 W Xe lamp (λ > 420 nm)	FeOOH QDs 2 wt%	10 ml IPA	224.24 μmol h^−1^ •g	(Ⅱ)	Zhang et al. (2020b)

^∗^ The reaction mechanism is direct two electron oxygen reduction reaction, The reaction formula is: O_2_ + 2e^−^ + 2H^+^ → H_2_O_2_ (Ⅰ); Step by step one electron oxygen reduction reaction, the reaction formula is: O_2_ + e^−^ + H^+^ → •OOH, •OOH + e^−^ + H^+^ → H_2_O_2_ (Ⅱ); OH^−^ + OH^−^→H_2_O_2_ (Ⅲ).

## 3 Effect of surface modification on photocatalytic activity

In addition to increasing the activity of H_2_O_2_ production by the deposition of co-catalysts, decreasing the decomposition rate *via* surface modification is essential to maximize the final concentration of H_2_O_2_.

### 3.1 Surface passivation modification

**FIGURE 3:**
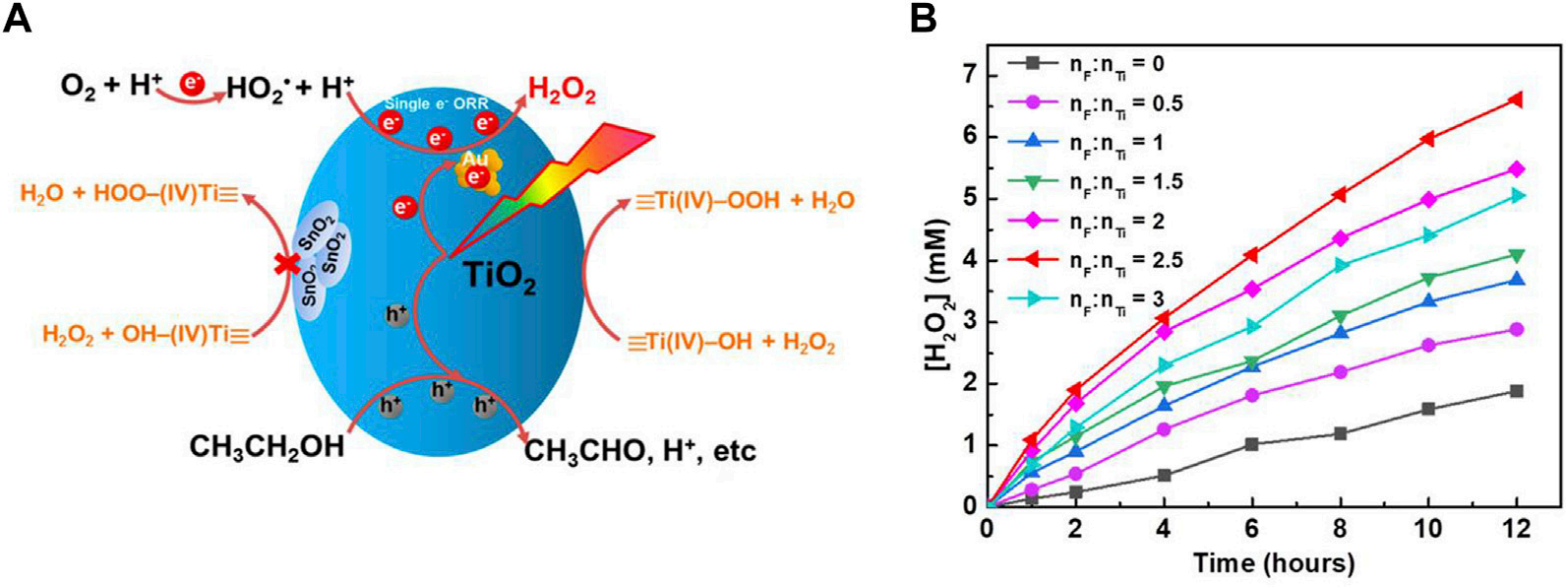
**(A)** Schematic illustration of H_2_O_2_ synthesis and decomposition over Au/SnO_2_ -TiO_2_ (Zuo et al., 2019b). **(B)** Photocatalytic H_2_O_2_ production over 0.1% Au/F-TiO_2_ prepared with different F/Ti ratios (Li L. et al., 2021).

TiO_2_ can catalyze the decomposition of H_2_O_2_ under visible light. Ti-OOH that form on the surface due to the interaction of TiO_2_ and H_2_O_2_ gradually degrade under visible light. This is the main reason for the decrease of H_2_O_2_ concentration during reactions (Li et al., 2001; Teranishi et al., 2010). Surface passivation can effectively inhibit the decomposition of H_2_O_2_. It can be carried out either by metal oxide passivation or non-metal passivation. Passivation of a photocatalyst with a metal oxide leads to the formation of a heterojunction (Zeng et al., 2017; Zuo et al., 2019b; Awa et al., 2020; Feng et al., 2021). For example, the surface of anatase TiO_2_ and rutile TiO_2_ were modified with SnO_2_ to form SnO_2_-TiO_2_ heterojunction. Then, the surface was functionalized with gold nanoparticles, and it was found that the formation activity of H_2_O_2_ was improved. This is because the decomposition of H_2_O_2_ on the TiO_2_ surface is inhibited (Figure 3A) (Zuo et al., 2019b). However, the H_2_O_2_ photocatalytic synthesis reaction rate of Au modified Bi_2_O_3_-TiO_2_ was better than that of Au/SnO_2_-TiO_2_. This is because not only the decomposition of H_2_O_2_ is inhibited, but also the carrier recombination in Bi_2_O_3_ is inhibited (Feng et al., 2021). A similar phenomenon is found in the heterojunction formed on g-C_3_N_4_ (Chen et al., 2017b; Chen X. L. et al., 2020; Liu et al., 2021b).

Non—metallic surface modification was also effective for improving photocatalytic activity (Maurino et al., 2005; Moon et al., 2014; Zhang M. M. et al., 2020; Li L. et al., 2021). For example, by hydrothermal treatment of TiO_2_ and NaF to obtain F-TiO_2_, the decomposition of H_2_O_2_ is inhibited. This was due to the fact that the F ion fixed on the TiO_2_ surface competes with the Ti-OOH formation, thus reducing the Ti-OOH formation. Therefore, it was no longer necessary to add NaF to the photocatalytic reaction medium (Figure 3B) (Li L. et al., 2021).

### 3.2 Organic molecular modification

**FIGURE 4:**
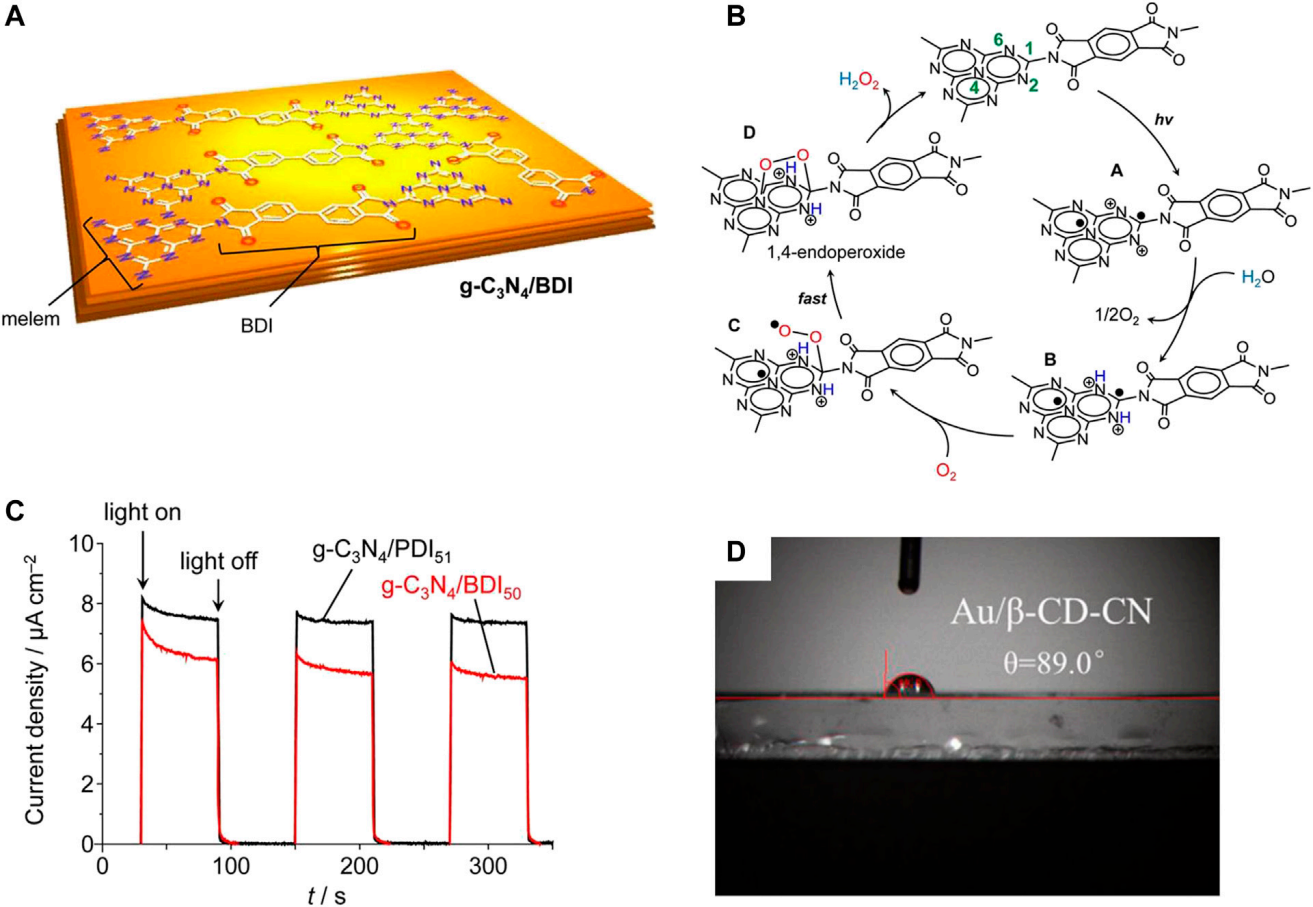
**(A)** Three-dimensional structure of g-C_3_N_4_/BDI. **(B)** Proposed Mechanism for H_2_O_2_ Formation on the Photoexcited g-C_3_N_4_/BDI Catalyst. **(C)** Photocurrent response of g-C_3_N_4_/BDI_50_ and g-C_3_N_4_/PDI_51_ in 0.1 M Na_2_SO_4_ solution under visible light (λ >420 nm) at a bias of 0.5 V vs. Ag/AgCl (Kofuji et al., 2016). **(D)** The water contact angle of Au/β-CD-CN (Zuo et al., 2020).

Imine organic molecules can be used to modify the surface of g-C_3_N_4_ to inhibit the electron-hole pair recombination of g-C_3_N_4_ (Shiraishi et al., 2014a; Kofuji et al., 2016; Yang et al., 2017; Goclon and Winkler, 2018; Guo et al., 2020; Zeng et al., 2020). For example, modification of the surface of g-C_3_N_4_ with homobendiimide and bibendiimide increased the synthesis activity of H_2_O_2_ (Figures 4A–C) (Shiraishi et al., 2014a; Kofuji et al., 2016). Besides the pure g-C_3_N_4_ reaction, another H_2_O_2_ synthesis pathway (•OH +•OH→H_2_O_2_) also plays a key role in the polyimide modified g-C_3_N_4_ nanosheets (Yang et al., 2017). In another publication, it was found that the modification of g-C_3_N_4_ by β-cyclodextrin can increase its hydrophobicity and affinity for oxygen, thus increasing the yield of H_2_O_2_ (Figure 4D) (Zuo et al., 2020).

Metal organic frameworks (MOFs) are promising materials that can be used to modify photocatalysts. This is because metal nodes and organic linkers of MOFs can be easily modified to improve photon absorption and catalytic activity. Therefore, various modification strategies have been devised, such as double substrate metal-organic framework, metal nanoparticles and MOF composite, etc (Wang Z. et al., 2020; Duan et al., 2020; Younis et al., 2020; Fang et al., 2021). The results showed that the activity of H_2_O_2_ synthesis was improved by modification of ZIF (Chang et al., 2020) and MIL (Isaka et al., 2019) type metal-organic framework materials. It was mainly attributed to the wider bandgap energy. Titanium-zirconium MOFs were prepared and used for photocatalytic production of H_2_O_2_ in two phase system (water/benzoic acid). Ti species effectively promoted electron transfer from the photoexcited linkers of MOFs to Ti and inhibited the recombination of electron-hole pairs in the hydrophobic MOFs matrix (Chen et al., 2020c). Table 2 summarizes the effects of different surface modifications on hydrogen peroxide production activity.

**TABLE 2:** Activities of photocatalysts with different types of surface modification.

Photocatalyst	Catalyst mass	Incident light	Load	Reaction condition	H_2_O_2_ activity	Function of modification	Reaction mechanism^∗^	References
M_V_-M_S_-CN/MAFO	0.2 g (200 ml)	250 W high-pressure sodium lamp (400–800 nm)	n_Mg_: n_Al_: n_Fe_ = 5 : 2: 1	O_2_, 0.5 mol L^−1^ NaNO_2_	6.3 mmol L^−1^ (18 h)	Surface passivation modification	(Ⅰ)	Chen et al. (2017b)
Au/SnO_2_-NR#TiO_2_	10 mg (10 ml)	300 W Xe lamp (λ > 430 nm)	—	O_2_, 4% EtOH	60 μM (6 h)	Surface passivation modification	(Ⅰ)	Awa et al. (2020)
F/TiO_2_(P25)	0.5 g/L	40 W fluorescent lamp (λ > 360 nm)	F 1.0 × 10^–2^ M	pH = 3.2, Air, 1.0 × 10^–2^ M HCOOH	1.3 × 10^–3^ mol•L^−1^ (100 min)	Surface passivation modification	(Ⅱ)	Maurino et al. (2005)
Au/SnO_2_-TiO_2_	0.2 g (200 ml)	300 W Xe arc lamp	Au 0.1 wt% n _Sn_:n_Ti_ = 4%	O_2_, 4% EtOH	9 mM (12 h)	Surface passivation modification	(Ⅱ)	Zuo et al. (2019b)
TiO_2_/rGO/WO_3_(TRW)	3 mg (30 ml)	200 W arc Mercury-Xenon research lamp	Na_2_WO_4_ 0.5 M	—	350 μM (80 min)	Surface passivation modification	(Ⅰ)	Zeng et al. (2017)
rGO/TiO_2_/P	0.5 g/L	λ > 320 nm	rGO 6 wt%, 0.1 M of phosphate buffer	Ph = 3, O_2_, 5 vol% IPA	4.5 mM (200 min)	Surface passivation modification	—	Moon et al. (2014)
HTNT-CD	20 mg (15 ml)	350 W Xe lamp (λ > 365 nm)	CDs 2.6 wt%	Air	3.42 mmol gcat^−1^ h^−1^	Quantum dots	(Ⅰ) (Ⅲ)	Ma et al. (2019)
g-C_3_N_4_/BDI	100 mg (30 ml)	λ > 420 nm	melem:BTCDA (mol:mol) = 1::2.5	O_2_, 10 vol% 2-PrOH	41 μmol (48 h)	Organic molecular modification	(Ⅰ)	Kofuji et al. (2016)
g-C_3_N_4_/PDI	50 mg (30 ml)	Xe lamp (λ > 420 nm)	—	O_2_	50.6 μmol (48 h)	Organic molecular modification	(Ⅱ)	Shiraishi et al. (2014a)
RF523 @333 K	50 mg (30 ml)	Xe lamp (λ > 420 nm)	—	O_2_	100 μmol (24 h)	Organic molecular modification	(Ⅰ)	Shiraishi et al. (2019)
PCNBA0.2	50 mg (30 ml)	500 W Xe lamp (λ > 420 nm)	melem:BA = 3 g: 0.2 g	O_2_	>2 mg/L (1 h)	Organic molecular modification	(Ⅱ)	Teng et al. (2020)
rGO/Cd_3_ (TMT)_2_	80 mg (20 ml)	λ > 420 nm	rGO 0.1 wt%	O_2_, 5 vol% MeOH	7 mmol•L^−1^ (24 h)	Organic molecular modification	(Ⅱ)	Xu et al. (2017)
PI-NCN	50 mg (50 ml)	300 W Xe lamp (λ > 420 nm)	PI 5 wt%	—	120 μmol (120 min)	Organic molecular modification	(Ⅱ) (Ⅲ)	Yang et al. (2017)
PEI/C_3_N_4_	20 mg (20 ml)	arc Xenon research lamp (Newport) with AM 1.5 air filter	PEI 50% W/V	O_2_	208.1 μM (60 min)	Organic molecular modification	(Ⅱ)	Zeng et al. (2020)
MIL-125-R7	5 mg (7 ml)	λ > 420 nm	caprylic anhydride treatment	BA/H_2_O = 5ml/2 ml	1500 μM (2 h)	MOF	(Ⅱ)	Isaka et al. (2019)
ZIF-8	0.05 g (100 ml)	350 W Xenon lamp	—	O_2_, water	75 μmol•L^−1^•h^−1^	MOF	—	Chang et al. (2020)
OPA/Zr_100-x_Ti_x_-MOF	5 mg (7 ml)	500 W Xe lamp (λ > 420 nm)	Ti: (Ti + Zr) (mol:mol) = 7.5%	O_2_, BA/H_2_O = 5ml/2 ml	9.7 mmol•L^−1^•h^−1^	MOF	(Ⅱ)	Chen et al. (2020c)

^∗^ The reaction mechanism is direct two electron oxygen reduction reaction, The reaction formula is: O_2_ + 2e^−^ + 2H^+^ → H_2_O_2_ (Ⅰ); Step by step one electron oxygen reduction reaction, the reaction formula is: O_2_ + e^−^ + H^+^ → •OOH, •OOH + e^−^ + H^+^ → H_2_O_2_ (Ⅱ); OH^−^ + OH^−^→H_2_O_2_ (Ⅲ).

## 4 Effect of doping on the photocatalytic activity

Doping elements can effectively reduce the band gap of photocatalysts to improve the utilization of solar light (Akpan and Hameed, 2010; Zhao et al., 2017). Studies have shown that doping can change the number of active sites, reduce the formation energy of •OOH intermediates, and promote the formation of H_2_O_2_ (Li X. et al., 2021). Therefore, incorporating metal and non-metal ions in the photocatalyst can improve the photocatalytic synthesis activity of H_2_O_2_ (Table 3).

**TABLE 3:** Activities of photocatalysts with different types of ion doping.

Photocatalyst	Catalyst mass	Incident light	Load	Reaction condition	H_2_O_2_ activity	Reaction pathway^∗^	References
KBH_4_/g-C_3_N_4_	50 mg (100 ml)	300 W Xe lamp (λ > 420 nm)	KBH_4_ 0.17 wt%	O_2_, 10 vol% IPA	287 μmol h^−1^	(Ⅱ)	Feng et al. (2020)
KPF_6_-CN	0.5 g/L	300 W Xe lamp (λ > 420 nm)	15 mmol KPF_6_	Ph = 3, O_2_, 10 vol% EtOH	1.5 mM (5 h)	(Ⅰ)	Kim et al. (2018c)
Cv-g-C_3_N_4_	0.1 g (100 ml)	300 W Xe lamp (λ > 420 nm)	—	O_2_	90 μM (60 min)	(Ⅰ)	Li et al. (2016)
K^+^-Na^+^/g-C_3_N_4_	0.2 g (200 ml)	250 W high-pressure sodium lamp (400–800 nm)	K^+^ 1.3 wt%, Na^+^ 0.7 wt%	O_2_, NaNO_2_ (0.5 mol L^−1^)	4.6 mmol L^−1^ (18 h)	(Ⅰ) (Ⅲ)	Qu et al. (2018)
Pt-KCN	0.2 g (200 ml)	250 W high-pressure sodium lamp (400–800 nm)	Pt 1 wt%, 5 ml KOH (0.1 mol/L)	Remove the air	620 μmol •g^−1^	(Ⅱ)	Hu et al. (2020)
K_2_HPO_4_/GCN	0.1 g (100 ml)	300 W Xe lamp (λ > 420 nm)	Urea (g): Dopant (mmol) = 10 : 10	O_2_, 10 vol% EtOH	5.05 mM (10 h)	(Ⅰ)	Tian et al. (2019)
OCN(24)	0.2 g (200 ml)	250 W high-pressure sodium lamp (400–800 nm)	—	O_2_, 0.5 mol L^−1^ NaNO_2_	3.8 mmol L^−1^ (12 h)	(Ⅰ) (Ⅲ)	Wang et al. (2019a)
Ni-FCN	0.2 g (200 ml)	250 W high-pressure sodium lamp (400–800 nm)	n_Ni_/dicyandiamide = 0.006	O_2_	7.7 mmol L^−1^ (12 h)	(Ⅰ)	Wu et al. (2017)
KPD-CN	20 mg (40 ml)	300 W Xe lamp (λ > 420 nm)	Urea (g): Dopant (mmol) = 4 : 7.5	pH = 3, O_2_, 10 vol% EtOH	1.5 mM (7 h)	(Ⅰ)	Moon et al. (2017)
rGO/TiO_2_/P	0.5 g/L	λ > 320 nm	rGO 6 wt%, 0.1 M of phosphate buffer	Ph = 3, O_2_, 5 vol% IPA	4.5 mM (200 min)	—	Moon et al. (2014)
OCN-500	—	λ > 420 nm	—	O_2_, 10 vol% IPA	730 μmol (5 h)	(Ⅰ)	Wei et al. (2018)
AQ/U-POCN	10 mg (20 ml)	300 W Xe lamp (400–780 nm)	U-POCN: AQ = 12 μM: 4 μM	Air	75 μM h^−1^	(Ⅱ)	Ye et al. (2021)
Br-H-GCN	0.2 g (200 ml)	250 W high-pressure sodium lamp (400–800 nm)	Br 0.75 wt%	O_2_, 0.15g EDTA	1.99 mmol L^−1^ (5 h)	(Ⅰ)	Zhang et al. (2018)

^∗^ The reaction mechanism is direct two electron oxygen reduction reaction, The reaction formula is: O_2_ + 2e^−^ + 2H^+^ → H_2_O_2_ (Ⅰ); Step by step one electron oxygen reduction reaction, the reaction formula is: O_2_ + e^−^ + H^+^ → •OOH, •OOH + e^−^ + H^+^ → H_2_O_2_ (Ⅱ); OH^−^ + OH^−^→H_2_O_2_ (Ⅲ).

### 4.1 Metal ion incorporation

**FIGURE 5:**
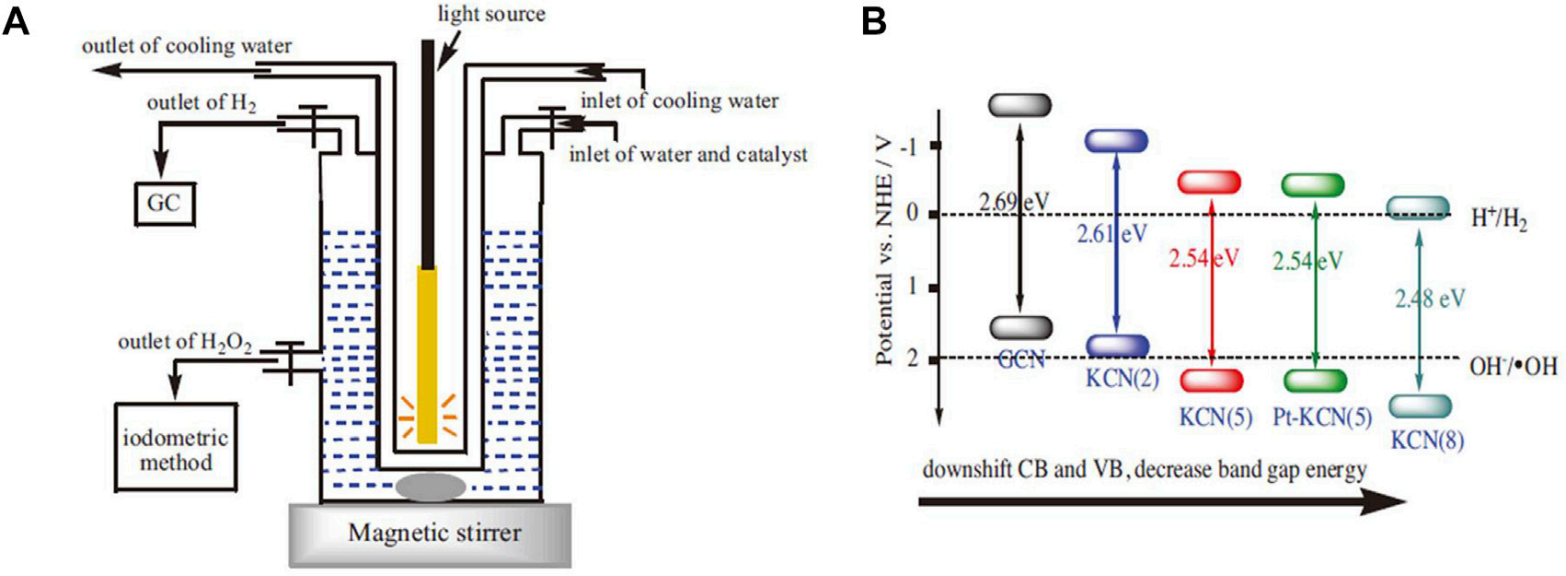
**(A)** The schematic diagram of the reactor. **(B)** The band position of K^+^/g-C_3_N_4_ (Hu et al., 2020).

Incorporating metal ions in the photocatalyst can improve the photocatalytic synthesis activity of H_2_O_2_ (Wu et al., 2017; Kim S. et al., 2018; Qu et al., 2018; Feng et al., 2020; Hu et al., 2020). For example, incorporating g-C_3_N_4_ with K^+^ can be used to photocatalyze water decomposition to produce H_2_ and H_2_O_2_ simultaneously without any sacrificial agent (Figure 5A). K^+^ was coordinated into the big C-N rings by forming the N-bridge, which inhibits the crystal growth of g-C_3_N_4_, promotes the specific surface area, increases the visible light absorption. More importantly, the CB and VB can be adjusted to the best position (Figure 5B) (Hu et al., 2020). A similar phenomenon of band gap adjustment was observed in g-C_3_N_4_ co-incorporated with K^+^and Na^+^. After the band gap adjustment, not only CB electrons can reduce O_2_ to produce H_2_O_2_, but also VB holes can oxidize OH^−^ to •OH for H_2_O_2_ synthesis. This made the generation mechanism of photocatalytic H_2_O_2_ change from “single channel pathway” (O_2_ + 2e^−^ + 2H^+^ → H_2_O_2_) to “dual channel pathway” (O_2_ + 2e^−^ + 2H^+^ → H_2_O_2_ and •OH+•OH→H_2_O_2_ reaction pathways) (Qu et al., 2018).

### 4.2 Non-metal ion doping

**FIGURE 6:**
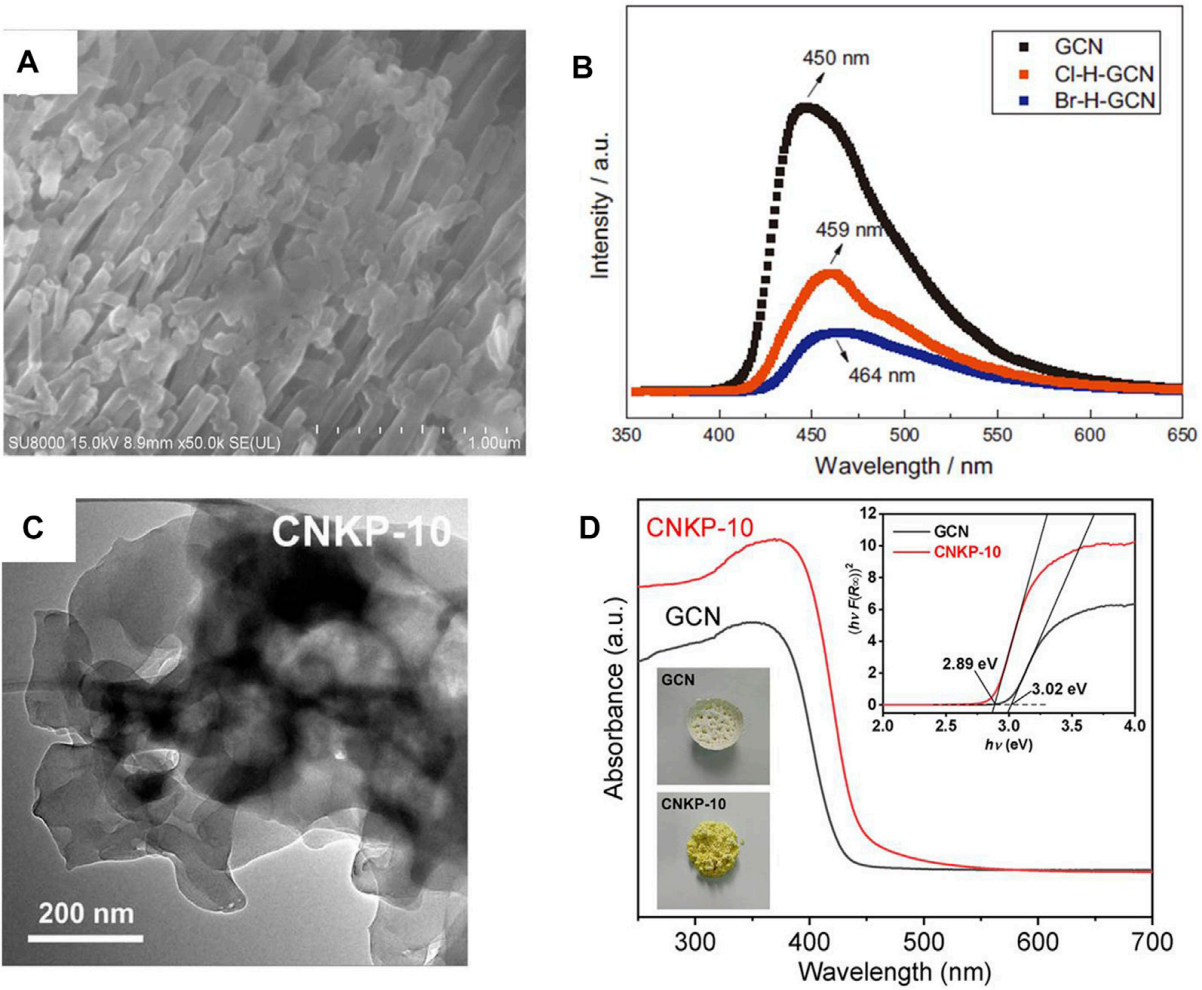
**(A)** The SEM images of Br-H-GCN. **(B)** PL spectra of GCN, Cl-H-GCN and Br-H-GCN (Zhang et al., 2018). **(C)** TEM images of the CNKP-10 catalysts. **(D)** UV–vis DRS spectra of the GCN and CNKP-10 catalysts (Tian et al., 2019).

Non-metal ion doping photocatalyst can effectively improve the synthetic activity of H_2_O_2_ (Moon et al., 2014; Wei et al., 2018; Zhang et al., 2018; Wang L. C. et al., 2019; Ye et al., 2021). For example, halogens (Cl and Br) were incorporated into g-C_3_N_4_ by hydrothermal method (Figure 6A), and it was found that g-C_3_N_4_ incorporated with Br was more conducive to H_2_O_2_ synthesis. This is mainly due to the larger specific surface area and higher charge separation rate after incorporating (Figure 6B) (Zhang et al., 2018). A similar phenomenon was observed in the co-doping of metal ions and non-metals (K and P) (Figure 6C). Compared with g-C_3_N_4_ incorporated with P (NH_4_H_2_PO_4_/GCN) or K^+^ (K_2_SO_4_/GCN), the H_2_O_2_ generation of g-C_3_N_4_ after co-incorporating was 10.98 times of the former and 5.2 times of the latter, respectively (Figure 6D) (Tian et al., 2019). Similarly, co-doping can also improve the catalytic activity of TiO_2_. For example, Fe and S were co-doped into TiO_2_ by one-step anodic oxidation, and it was found that the synthesis activity of TiO_2_ was improved after doping. This is mainly attributed to the fact that Fe-S co doped TiO_2_ had a narrower band gap than pure TiO_2_, resulting in a wider visible light absorption range (Momeni and Akbarnia, 2021).

## 5 Effect of reaction environment on photocatalytic activity

### 5.1 Effects of temperature and pH

One study investigated the effect of temperature and pH on the photoactivity of H_2_O_2_ generation by using Au/TiO_2_ photocatalyst. The results showed that when pH value (pH = 2) or temperature (5°C) was low, it was more beneficial to improve the photoactivity. The main reason was that the thermal catalytic decomposition of H_2_O_2_ by Au/TiO_2_ can be effectively inhibited at low pH value or low temperature (Teranishi et al., 2016). In another study, it was found that low pH also increased the H_2_O_2_ synthesis activity of MOFs materials. At the same temperature, when the pH value of MOFs material was as low as 0.3, the formation of H_2_O_2_ was more favorable. (Isaka et al., 2019).

### 5.2 Effects of sacrificial agents

For the photocatalytic production of H_2_O_2_, a certain amount of sacrificial agent is usually added to act as hole scavenger and prevent the recombination of electron–hole pairs. The sacrificial agents were mainly alcohols, which provided hydrogen source for photocatalytic H_2_O_2_ generation (Kormann et al.). However, the ability of aliphatic alcohols (such as ethanol and methanol, which act as electron donors) to improve photoactivity is limited. The results showed that g-C_3_N_4_ can effectively synthesize H_2_O_2_ in deionized water containing oxygen under visible light irradiation. This was due to the efficient formation of 1, 4-endoperoxide on the surface of g-C_3_N_4_. The addition of ethanol inhibited the one-electron reduction of O_2_ (formation of superoxide radicals) and selectively promoted the two-electron reduction of O_2_. At the same time, the photodecomposition of hydrogen peroxide formed subsequently was inhibited (Shiraishi et al., 2014b). Kim et al, (2016) also investigated whether the addition of electron donors affects photocatalytic activity. The results showed that when no sacrificial agent (methanol) was added to the system, the generation activity of H_2_O_2_ was extremely low. This result confirmed that using sacrificial agents such as methanol is important. When methanol (5 vol%) was present in the system, H_2_O_2_ was generated together with formaldehyde (CH_3_OH + O_2_ → HCHO + H_2_O_2_). Meanwhile, ethanol and 2-propanol were tested further, and the results showed that these alcohols worked effectively as electron donors.

**FIGURE 7:**
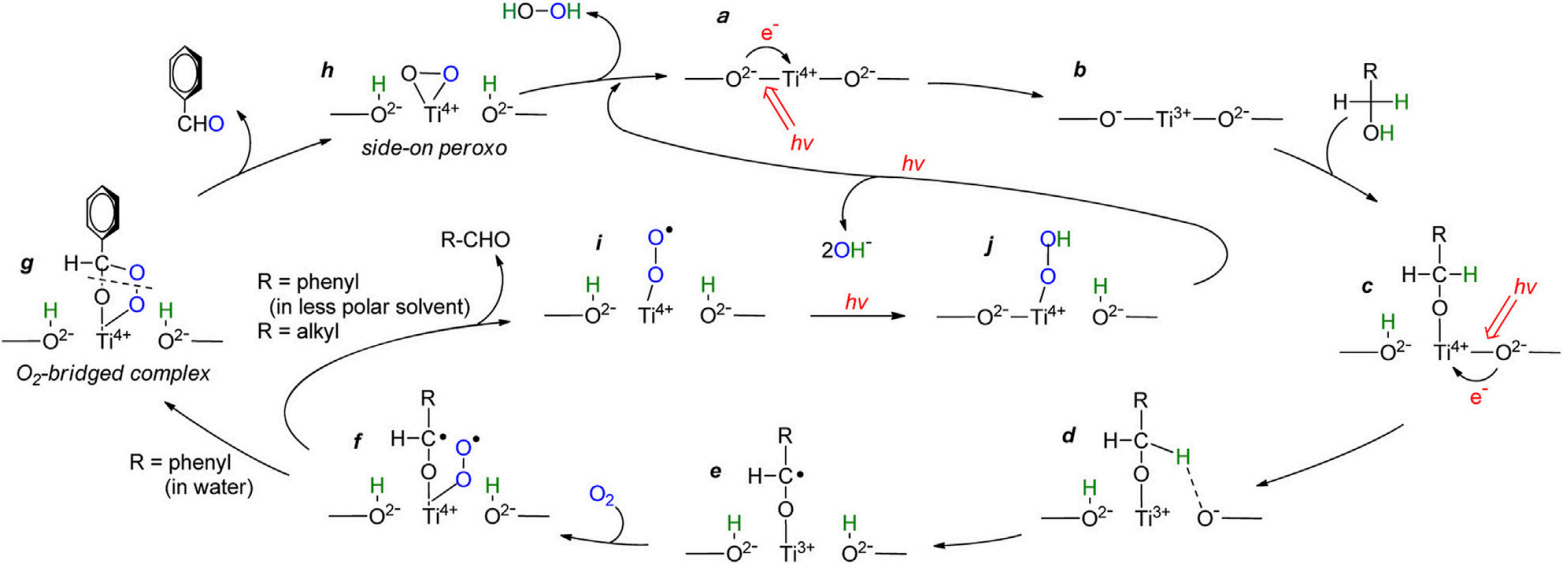
Proposed mechanism for photocatalytic oxidation of alcohols with O_2_ on the TiO_2_ Surface (Shiraishi et al., 2013).

However, aliphatic alcohols (such as ethanol and methanol) as electron donors have limited improvement in photocatalytic activity for hydrogen peroxide synthesis. Therefore, some studies have tried to use aromatic alcohol (benzyl alcohol) as a sacrifice agent, compared with fatty alcohol. The results showed that, during photoreaction with aliphatic alcohol, the carbon radical was rapidly removed and leaved superoxo radical (the f→i process in Figure 7), resulting in very low for H_2_O_2_ formation. In the aqueous phase containing benzyl alcohol, the carbon free radical was stably transformed into an oxygen bridge complex (f→g→h process in Figure 7), which generates a large number of peroxides and improves the synthesis activity of H_2_O_2_. The results showed that benzyl alcohol as electron donor can improve the reactivity. (Shiraishi et al., 2013).

## Conclusions and outlook

In future, the photocatalytic H_2_O_2_ synthesis system still need to improve the reaction activity and sustainability. We should consistently increase the upper limit of H_2_O_2_ production concentration in long-term photocatalytic reaction. The kinetics of photocatalytic H_2_O_2_ decomposition should be especially concerned. It is also urgent to develop efficient non-precious cocatalysts with two-electron ORR selectivity.

The activity of H_2_O_2_ synthesis was very unsatisfactory in most pure water systems. As a compromise for adding sacrificial reagents, the photocatalytic H_2_O_2_ synthesis system could be coupled with other valuable photocatalytic selective oxidation reaction to maximize its value, such as coupling with selective oxidation or photocatalytic degradation reactions.

## References

[B1] Adams J. S. Kromer M. L. Rodriguez-Lopez J. Flaherty D. W. (2021). Unifying concepts in electro- and thermocatalysis toward hydrogen peroxide production. J. Am. Chem. Soc. 143, 7940–7957. 10.1021/jacs.0c13399 34019397

[B2] Akpan U. G. Hameed B. H. (2010). The advancements in sol–gel method of doped-TiO_2_ photocatalysts. Appl. Catal. A General 375, 1–11. 10.1016/j.apcata.2009.12.023

[B3] Anantharaj S. Pitchaimuthu S. Noda S. (2021). A review on recent developments in electrochemical hydrogen peroxide synthesis with a critical assessment of perspectives and strategies. Adv. Colloid Interface Sci. 287, 102331. 10.1016/j.cis.2020.102331 33321333

[B4] Andersen B. M. Rasch M. Hochlin K. Jensen F. H. Wismar P. Fredriksen J. E. (2006). Decontamination of rooms, medical equipment and ambulances using an aerosol of hydrogen peroxide disinfectant. J. Hosp. Infect. 62, 149–155. 10.1016/j.jhin.2005.07.020 16337307 PMC7114946

[B5] Apaydin D. H. Seelajaroen H. Pengsakul O. Thamyongkit P. Sariciftci N. S. Kunze-Liebhauser J. (2018). Photoelectrocatalytic synthesis of hydrogen peroxide by molecular copper-porphyrin supported on titanium dioxide nanotubes. ChemCatChem 10, 1793–1797. 10.1002/cctc.201702055 29780435 PMC5947148

[B6] Awa K. Naya S.-I. Fujishima M. Tada H. (2020). A three-component plasmonic photocatalyst consisting of gold nanoparticle and TiO_2_–SnO_2_ nanohybrid with heteroepitaxial junction: Hydrogen peroxide synthesis. J. Phys. Chem. C 124, 7797–7802. 10.1021/acs.jpcc.9b11875

[B7] Baran T. Wojtyla S. Vertova A. Minguzzi A. Rondinini S. (2018). Photoelectrochemical and photocatalytic systems based on titanates for hydrogen peroxide formation. J. Electroanal. Chem. 808, 395–402. 10.1016/j.jelechem.2017.06.044

[B8] Cai J. Huang J. Wang S. Iocozzia J. Sun Z. Sun J. (2019). Crafting mussel-inspired metal nanoparticle-decorated ultrathin graphitic carbon nitride for the degradation of chemical pollutants and production of chemical resources. Adv. Mater 31, e1806314. 10.1002/adma.201806314 30697837

[B9] Campos-Martin J. M. Blanco-Brieva G. Fierro J. L. (2006). Hydrogen peroxide synthesis: An outlook beyond the anthraquinone process. Angew. Chem. Int. Ed. Engl. 45, 6962–6984. 10.1002/anie.200503779 17039551

[B10] Chang A. L. Nguyen V. H. Lin K. Y. A. Hu C. C. (2020). Selective synthesis of ZIFs from zinc and nickel nitrate solution for photocatalytic H_2_O_2_ production. Arabian J. Chem. 13, 8301–8308. 10.1016/j.arabjc.2020.04.027

[B11] Chang S. L. Li H. Liu J. N. Zhao M. X. Tan M. H. Xu P. W. (2021). Effect of hydrogen peroxide treatment on the quality of epsilon-poly-L-lysine products. Biochem. Eng. J. 171, 108017. 10.1016/j.bej.2021.108017

[B12] Chang X. Y. Yang J. J. Han D. D. Zhang B. Xiang X. He J. (2018). Enhancing light-driven production of hydrogen peroxide by anchoring Au onto C_3_N_4_ catalysts. Catalysts 8, 147. 10.3390/catal8040147

[B13] Chen J. Y. Cao J. M. Zhou J. Wang W. Q. Zhang Y. F. Liu J. F. (2020a). A computational evaluation of MoS_2_-based materials for the electrocatalytic oxygen reduction reaction. New J. Chem. 44, 14189–14197. 10.1039/d0nj02621b

[B14] Chen Q. (2008). Development of an anthraquinone process for the production of hydrogen peroxide in a trickle bed reactor—from bench scale to industrial scale. Chem. Eng. Process. Process Intensif. 47, 787–792. 10.1016/j.cep.2006.12.012

[B15] Chen S. Tu R. Li J. Lu X. (2018). Pd catalysts supported on rGO-TiO_2_ composites for direct synthesis of H_2_O_2_ : Modification of Pd^2+^/Pd^0^ ratio and hydrophilic property. Chin. J. Chem. Eng. 26, 534–539. 10.1016/j.cjche.2017.07.016

[B16] Chen X. Hu S. Z. Li P. Li W. Ma H. F. Lu G. (2017). Photocatalytic production of hydrogen peroxide using g-C_3_N_4_ coated MgO-Al_2_O_3_-Fe_2_O_3_ heterojunction catalysts prepared by a novel molten salt-assisted microwave process. Acta Physico-Chimica Sin. 33, 2532–2541. 10.3866/pku.Whxb201706153

[B17] Chen X. L. Kuwahara Y. Mori K. Louis C. Yamashita H. (2020b). A hydrophobic titanium doped zirconium-based metal organic framework for photocatalytic hydrogen peroxide production in a two-phase system. J. Mater. Chem. A 8, 1904–1910. 10.1039/c9ta11120d

[B18] Chen X. Zhang W. Zhang L. Feng L. Zhang C. Jiang J. (2020). Sacrificial agent-free photocatalytic H_2_O_2_ evolutionviatwo-electron oxygen reduction using a ternary α-Fe_2_O_3_/CQD@g-C_3_N_4_ photocatalyst with broad-spectrum response. J. Mater. Chem. A 8, 18816–18825. 10.1039/d0ta05753c

[B19] Chen Y. M. Gu W. Q. Tan L. Ao Z. M. An T. C. Wang S. B. (2021). Photocatalytic H_2_O_2_ production using Ti_3_C_2_ MXene as a non-noble metal cocatalyst. Appl. Catal. a-General 618, 118127. 10.1016/j.apcata.2021.118127

[B20] Chen Z. Li C. Ni Y. Kong F. Kong A. Shan Y. (2017). Ag-Enhanced catalytic performance of ordered mesoporous Fe–N-graphitic carbons for oxygen electroreduction. Catal. Lett. 147, 2745–2754. 10.1007/s10562-017-2186-2

[B21] Chung S. Chung J. Chung C. (2020). Enhanced electrochemical oxidation process with hydrogen peroxide pretreatment for removal of high strength ammonia from semiconductor wastewater. J. Water Process Eng. 37, 101425. 10.1016/j.jwpe.2020.101425

[B22] Dinakar M. Tao W. D. Daley D. (2020). Using hydrogen peroxide to supplement oxygen for nitrogen removal in constructed wetlands. J. Environ. Chem. Eng. 8, 104517. 10.1016/j.jece.2020.104517

[B23] Du L. Zhang G. X. Liu X. H. Hassanpour A. Dubois M. Tavares A. C. (2020). Biomass-derived nonprecious metal catalysts for oxygen reduction reaction: The demand-oriented engineering of active sites and structures. Carbon Energy 2, 561–581. 10.1002/cey2.73

[B24] Duan M. B. Jiang L. B. Zeng G. M. Wang D. B. Tang W. W. Liang J. (2020). Bimetallic nanoparticles/metal-organic frameworks: Synthesis, applications and challenges. Appl. Mater. Today 19, 100564. 10.1016/j.apmt.2020.100564

[B25] Edwards J. K. Hutchings G. J. (2008). Palladium and gold-palladium catalysts for the direct synthesis of hydrogen peroxide. Angew. Chem. Int. Ed. Engl. 47, 9192–9198. 10.1002/anie.200802818 18798185

[B26] Fang Y. Yang Y. Yang Z. G. Li H. P. Roesky H. W. (2021). Advances in design of metal-organic frameworks activating persulfate for water decontamination. J. Organomet. Chem. 954, 122070. 10.1016/j.jorganchem.2021.122070

[B27] Feng C. Y. Tang L. Deng Y. C. Wang J. J. Luo J. Liu Y. N. (2020). Synthesis of leaf-vein-like g-C(3)N(4)with tunable band structures and charge transfer properties for selective photocatalytic H(2)O(2)Evolution. Adv. Funct. Mater. 30, 2001922. 10.1002/adfm.202001922

[B28] Feng L. W. Li B. D. Xiao Y. Q. Li L. J. Zhang Y. Q. Zhao Q. N. (2021). Au modified Bi_2_O_3_-TiO_2_ hybrid for photocatalytic synthesis of hydrogen peroxide. Catal. Commun. 155, 106315. 10.1016/j.catcom.2021.106315

[B29] Fukuzumi S. Lee Y. M. Nam W. (2018). Solar-Driven production of hydrogen peroxide from water and dioxygen. Chemistry 24, 5016–5031. 10.1002/chem.201704512 29105181

[B30] Gao G. H. Tian Y. N. Gong X. X. Pan Z. Y. Yang K. Y. Zong B. N. (2020). Advances in the production technology of hydrogen peroxide. Chin. J. Catal. 41, 1039–1047. 10.1016/S1872-2067(20)63562-8

[B31] Goclon J. Winkler K. (2018). Computational insight into the mechanism of O_2_ to H_2_O_2_ reduction on amino-groups-containing g-C_3_N_4_ . Appl. Surf. Sci. 462, 134–141. 10.1016/j.apsusc.2018.08.070

[B32] Guarino V. A. Oldham W. M. Loscalzo J. Zhang Y. Y. (2019). Reaction rate of pyruvate and hydrogen peroxide: Assessing antioxidant capacity of pyruvate under biological conditions. Sci. Rep. 9, 19568. 10.1038/s41598-019-55951-9 31862934 PMC6925109

[B33] Guo F. Zhang M. Yi S. Li X. Xin R. Yang M. (2022). Metal-coordinated porous polydopamine nanospheres derived Fe_3_N-FeCo encapsulated N-doped carbon as a highly efficient electrocatalyst for oxygen reduction reaction. Nano Res. Energy 1, e9120027. 10.26599/NRE.2022.9120027

[B34] Guo Y. Li H. R. Ma W. Shi W. X. Zhu Y. F. Choi W. Y. (2020). Photocatalytic activity enhanced via surface hybridization. Carbon Energy 2, 308–349. 10.1002/cey2.66

[B35] Haider Z. Cho H. I. Moon G. H. Kim H. I. (2019). Minireview: Selective production of hydrogen peroxide as a clean oxidant over structurally tailored carbon nitride photocatalysts. Catal. Today 335, 55–64. 10.1016/j.cattod.2018.11.067

[B36] Halder R. Lawal A. (2007). Experimental studies on hydrogenation of anthraquinone derivative in a microreactor. Catal. Today 125, 48–55. 10.1016/j.cattod.2007.03.055

[B37] Han Y. He Z. Y. Wang S. L. Li W. Zhang J. L. (2015). Performance of facet-controlled Pd nanocrystals in 2-ethylanthraquinone hydrogenation. Catal. Sci. Technol. 5, 2630–2639. 10.1039/c5cy00050e

[B38] Hirakawa H. Shiota S. Shiraishi Y. Sakamoto H. Ichikawa S. Hirai T. (2016). Au nanoparticles supported on BiVO_4_: Effective inorganic photocatalysts for H_2_O_2_ production from water and O_2_ under visible light. ACS Catal. 6, 4976–4982. 10.1021/acscatal.6b01187

[B39] Hu S. Sun X. Zhao Y. Li W. Wang H. Wu G. (2020). The effective photocatalytic water splitting to simultaneously produce H_2_ and H_2_O_2_ over Pt loaded K-g-C_3_N_4_ catalyst. J. Taiwan Inst. Chem. Eng. 107, 129–138. 10.1016/j.jtice.2019.12.007

[B40] Ignaczak A. Santos E. Schmickler W. (2019). Oxygen reduction reaction on gold in alkaline solutions - the inner or outer sphere mechanisms in the light of recent achievements. Curr. Opin. Electrochem. 14, 180–185. 10.1016/j.coelec.2018.07.011

[B41] Isaka Y. Kawase Y. Kuwahara Y. Mori K. Yamashita H. (2019). Two-phase system utilizing hydrophobic metal-organic frameworks (MOFs) for photocatalytic synthesis of hydrogen peroxide. Angew. Chem. Int. Ed. Engl. 58, 5402–5406. 10.1002/anie.201901961 30793452

[B42] Jeon T. Y. Yu S. H. Yoo S. J. Park H. Y. Kim S. K. (2020). Electrochemical determination of the degree of atomic surface roughness in Pt–Ni alloy nanocatalysts for oxygen reduction reaction. Carbon Energy 3, 375–383. 10.1002/cey2.82

[B43] Jia X. W. Sun F. Fei Y. Jin M. P. Zhang F. Xu W. (2018). Explosion characteristics of mixtures containing hydrogen peroxide and working solution in the anthraquinone route to hydrogen peroxide. Process Saf. Environ. Prot. 119, 218–222. 10.1016/j.psep.2018.08.007

[B44] Jirkovsky J. S. Halasa M. Schiffrin D. J. (2010). Kinetics of electrocatalytic reduction of oxygen and hydrogen peroxide on dispersed gold nanoparticles. Phys. Chem. Chem. Phys. 12, 8042–8052. 10.1039/c002416c 20505889

[B45] Jirkovsky J. S. Panas I. Ahlberg E. Halasa M. Romani S. Schiffrin D. J. (2011). Single atom hot-spots at Au-Pd nanoalloys for electrocatalytic H_2_O_2_ production. J. Am. Chem. Soc. 133, 19432–19441. 10.1021/ja206477z 22023652

[B46] Kim H. I. Choi Y. Hu S. Choi W. Kim J. H. (2018b). Photocatalytic hydrogen peroxide production by anthraquinone-augmented polymeric carbon nitride. Appl. Catal. B-Environmental 229, 121–129. 10.1016/j.apcatb.2018.01.060

[B47] Kim H. I. Kwon O. S. Kim S. Choi W. Kim J. H. (2016). Harnessing low energy photons (635 nm) for the production of H_2_O_2_ using upconversion nanohybrid photocatalysts. Energy & Environ. Sci. 9, 1063–1073. 10.1039/c5ee03115j

[B48] Kim J. Kim H. E. Lee H. (2018a). Single-atom catalysts of precious metals for electrochemical reactions. ChemSusChem 11, 104–113. 10.1002/cssc.201701306 28895315

[B49] Kim S. Moon G. H. Kim H. Mun Y. Zhang P. Lee J. (2018c). Selective charge transfer to dioxygen on KPF_6_-modified carbon nitride for photocatalytic synthesis of H_2_O_2_ under visible light. J. Catal. 357, 51–58. 10.1016/j.jcat.2017.10.002

[B50] Kofuji Y. Ohkita S. Shiraishi Y. Sakamoto H. Tanaka S. Ichikawa S. (2016). Graphitic carbon nitride doped with biphenyl diimide: Efficient photocatalyst for hydrogen peroxide production from water and molecular oxygen by sunlight. Acs Catal. 6, 7021–7029. 10.1021/acscatal.6b02367

[B51] Kormann C. Bahnemann D. W. Hoffmann M. R. (1988). Photocatalytic production of hydrogen peroxides and organic peroxides in aqueous suspensions of titanium dioxide, zinc oxide, and desert sand. Environ. Sci. Technol. 22, 798–806. 10.1021/es00172a009 22195664

[B52] Kozlova L. S. Novikov V. T. Garaeva G. R. Gol'din M. M. Kolesnikov V. A. (2015). Electrodes modified with carbon materials in electrosynthesis of the dissolved hydrogen peroxide solutions and their medical properties. Prot. Metals Phys. Chem. Surfaces 51, 985–989. 10.1134/S2070205115060131

[B53] Li L. Li B. Feng L. Zhang X. Zhang Y. Zhao Q. (2021a). Au modified F-TiO_2_ for efficient photocatalytic synthesis of hydrogen peroxide. Molecules 26, 3844. 10.3390/molecules26133844 34202599 PMC8270298

[B54] Li S. N. Dong G. H. Hailili R. Yang L. P. Li Y. X. Wang F. (2016). Effective photocatalytic H_2_O_2_ production under visible light irradiation at g-C_3_N_4_ modulated by carbon vacancies. Appl. Catal. B-Environmental 190, 26–35. 10.1016/j.apcatb.2016.03.004

[B55] Li X. Wang X. Xiao G. Zhu Y. (2021b). Identifying active sites of boron, nitrogen co-doped carbon materials for the oxygen reduction reaction to hydrogen peroxide. J. Colloid Interface Sci. 602, 799–809. 10.1016/j.jcis.2021.06.068 34171746

[B56] Li X. Z. Chen C. C. Zhao J. C. (2001). Mechanism of photodecomposition of H_2_O_2_ on TiO_2_ surfaces under visible light irradiation. Langmuir 17, 4118–4122. 10.1021/la010035s

[B57] Liu B. Bie C. Zhang Y. Wang L. Li Y. Yu J. (2021). Hierarchically porous ZnO/g-C_3_N_4_ S-scheme heterojunction photocatalyst for efficient H_2_O_2_ production. Langmuir 37, 14114–14124. 10.1021/acs.langmuir.1c02360 34808051

[B58] Liu Y. M. Roy S. Sarkar S. Xu J. Q. Zhao Y. F. Zhang J. J. (2021). A review of carbon dots and their composite materials for electrochemical energy technologies. Carbon Energy 3, 795–826. 10.1002/cey2.134

[B59] Ma R. Y. Wang L. Wang H. Liu Z. Y. Xing M. Y. Zhu L. F. (2019). Solid acids accelerate the photocatalytic hydrogen peroxide synthesis over a hybrid catalyst of titania nanotube with carbon dot. Appl. Catal. B-Environmental 244, 594–603. 10.1016/j.apcatb.2018.11.087

[B60] Maurino V. Minero C. Mariella G. Pelizzetti E. (2005). Sustained production of H_2_O_2_ on irradiated TiO_2_-fluoride systems. Chem. Commun. (Camb), 2627–2629. 10.1039/b418789j 15900349

[B61] Meng X. G. Zong P. X. Wang L. Yang F. Hou W. S. Zhang S. T. (2020). Au-nanoparticle-supported ZnO as highly efficient photocatalyst for H_2_O_2_ production. Catal. Commun. 134, 105860. 10.1016/j.catcom.2019.105860

[B62] Momeni M. M. Akbarnia M. (2021). Photoelectrochemical, photocatalytic and electrochemical hydrogen peroxide production using Fe/S-codoped TiO_2_ nanotubes as new visible-light-absorbing photocatalysts. Appl. Phys. A 127, 449. 10.1007/s00339-021-04574-x

[B63] Moon G.-H. Fujitsuka M. Kim S. Majima T. Wang X. Choi W. (2017). Eco-friendly photochemical production of H_2_O_2_ through O_2_ reduction over carbon nitride frameworks incorporated with multiple heteroelements. ACS Catal. 7, 2886–2895. 10.1021/acscatal.6b03334

[B64] Moon G.-H. Kim W. Bokare A. D. Sung N.-E. Choi W. (2014). Solar production of H_2_O_2_ on reduced graphene oxide–TiO_2_ hybrid photocatalysts consisting of earth-abundant elements only. Energy Environ. Sci. 7, 4023–4028. 10.1039/c4ee02757d

[B65] Moreno C. M. (2011). Hydrogen peroxide production driven by UV-B in planktonic microorganisms: A photocatalytic factor in sea warming and ice melting in regions with ozone depletion? Biogeochemistry 107, 1–8. 10.1007/s10533-010-9566-7

[B66] Noh J. H. Yoo S. H. Son H. Fish K. E. Douterelo I. Maeng S. K. (2020). Effects of phosphate and hydrogen peroxide on the performance of a biological activated carbon filter for enhanced biofiltration. J. Hazard Mater 388, 121778. 10.1016/j.jhazmat.2019.121778 31818662

[B67] Peng Y. L. Wang L. Z. Liu Y. D. Chen H. J. Lei J. Y. Zhang J. L. (2017). Visible-light-Driven photocatalytic H_2_O_2_ production on g-C_3_N_4_ loaded with CoP as a noble metal free cocatalyst. Eur. J. Inorg. Chem. 2017, 4797–4802. 10.1002/ejic.201700930

[B68] Qu X. Hu S. Bai J. Li P. Lu G. Kang X. (2018). Synthesis of band gap-tunable alkali metal modified graphitic carbon nitride with outstanding photocatalytic H_2_O_2_ production ability via molten salt method. J. Mater. Sci. Technol. 34, 1932–1938. 10.1016/j.jmst.2018.04.019

[B69] Shiraishi Y. Kanazawa S. Kofuji Y. Sakamoto H. Ichikawa S. Tanaka S. (2014a). Sunlight-driven hydrogen peroxide production from water and molecular oxygen by metal-free photocatalysts. Angew. Chem. Int. Ed. Engl. 53, 13454–13459. 10.1002/anie.201407938 25293501

[B70] Shiraishi Y. Kanazawa S. Sugano Y. Tsukamoto D. Sakamoto H. Ichikawa S. (2014b). Highly selective production of hydrogen peroxide on graphitic carbon nitride (g-C_3_N_4_) photocatalyst activated by visible light. Acs Catal. 4, 774–780. 10.1021/cs401208c

[B71] Shiraishi Y. Kanazawa S. Tsukamoto D. Shiro A. Sugano Y. Hirai T. (2013). Selective hydrogen peroxide formation by titanium dioxide photocatalysis with benzylic alcohols and molecular oxygen in water. Acs Catal. 3, 2222–2227. 10.1021/cs400511q

[B72] Shiraishi Y. Takii T. Hagi T. Mori S. Kofuji Y. Kitagawa Y. (2019). Resorcinol-formaldehyde resins as metal-free semiconductor photocatalysts for solar-to-hydrogen peroxide energy conversion. Nat. Mater 18, 985–993. 10.1038/s41563-019-0398-0 31263224

[B73] Shu C. Tan Q. Deng C. Du W. Gan Z. Liu Y. (2021). Hierarchically mesoporous carbon spheres coated with a single atomic Fe–N–C layer for balancing activity and mass transfer in fuel cells. Carbon Energy 4, 1–11. 10.1002/cey2.136

[B74] Song H. Y. Wei L. S. Chen C. X. Wen C. C. Han F. Q. (2019). Photocatalytic production of H_2_O_2_ and its *in situ* utilization over atomic-scale Au modified MoS_2_ nanosheets. J. Catal. 376, 198–208. 10.1016/j.jcat.2019.06.015

[B75] Sterenchuk T. P. Belykh L. B. Skripov N. I. Sanzhieva S. B. Gvozdovskaya K. L. Schmidt F. K. (2018). The effect of particle size and the modifier on the properties of palladium catalysts in the synthesis of hydrogen peroxide by the anthraquinone method. Kinet. Catal. 59, 585–592. 10.1134/S0023158418050166

[B76] Sun Z. Sheng L. Gong H. Song L. Jiang X. Wang S. (2020). Electrocatalytic synthesis of hydrogen peroxide over Au/TiO_2_ and electrochemical trace of OOH* intermediate. Chem. Asian J. 15, 4280–4285. 10.1002/asia.202001089 33089926

[B77] Teng Z. Y. Cai W. Liu S. X. Wang C. Y. Zhang Q. T. Su C. L. (2020). Bandgap engineering of polymetric carbon nitride copolymerized by 2, 5, 8-triamino-tri-s-triazine (melem) and barbituric acid for efficient nonsacrificial photocatalytic H_2_O_2_ production. Appl. Catal. B-Environmental 271, 118917. 10.1016/j.apcatb.2020.118917

[B78] Teranishi M. Kunimoto T. Naya S. Kobayashi H. Tada H. (2020). Visible-light-Driven hydrogen peroxide synthesis by a hybrid photocatalyst consisting of bismuth vanadate and bis(hexafluoroacetylacetonato)copper(II) complex. J. Phys. Chem. C 124, 3715–3721. 10.1021/acs.jpcc.9b11568

[B79] Teranishi M. Naya S. Tada H. (2010). *In situ* liquid phase synthesis of hydrogen peroxide from molecular oxygen using gold nanoparticle-loaded titanium(IV) dioxide photocatalyst. J. Am. Chem. Soc. 132, 7850–7851. 10.1021/ja102651g 20486700

[B80] Teranishi M. Naya S. Tada H. (2016). Temperature- and pH-dependence of hydrogen peroxide formation from molecular oxygen by gold nanoparticle-loaded titanium(IV) oxide photocatalyst. J. Phys. Chem. C 120, 1083–1088. 10.1021/acs.jpcc.5b10626

[B81] Tian J. Wu T. J. Wang D. Pei Y. Qiao M. H. Zong B. N. (2019). One-pot synthesis of potassium and phosphorus-doped carbon nitride catalyst derived from urea for highly efficient visible light-driven hydrogen peroxide production. Catal. Today 330, 171–178. 10.1016/j.cattod.2018.07.039

[B82] Tsukamoto D. Shiro A. Shiraishi Y. Sugano Y. Ichikawa S. Tanaka S. (2012). Photocatalytic H2O2 production from ethanol/O2 system using TiO2 loaded with Au–Ag bimetallic alloy nanoparticles. ACS Catal. 2, 599–603. 10.1021/cs2006873

[B83] Viswanathan V. Hansen H. A. Rossmeisl J. Norskov J. K. (2012). Unifying the 2e(-) and 4e(-) Reduction of Oxygen on Metal Surfaces. J. Phys. Chem. Lett. 3, 2948–2951. 10.1021/jz301476w 26292231

[B84] Wang H. Guan Y. Hu S. Pei Y. Ma W. Fan Z. (2019b). Hydrothermal synthesis of band gap-tunable oxygen-doped g-C_3_N_4_ with outstanding “two-channel” photocatalytic H_2_O_2_ production ability assisted by dissolution–precipitation process. Nano 14, 1950023. 10.1142/s1793292019500231

[B85] Wang L. C. Cao S. Guo K. Wu Z. J. Ma Z. Piao L. Y. (2019a). Simultaneous hydrogen and peroxide production by photocatalytic water splitting. Chin. J. Catal., 40, 470–475. 10.1016/S1872-2067(19)63274-2

[B86] Wang Y. Shi L. Zhu J. Li B. Jin Y. (2020a). Visual and sensitive detection of telomerase activity via hydrogen peroxide test strip. Biosens. Bioelectron. 156, 112132. 10.1016/j.bios.2020.112132 32174558

[B87] Wang Z. Huang J. Mao J. Guo Q. Chen Z. Lai Y. (2020b). Metal–organic frameworks and their derivatives with graphene composites: Preparation and applications in electrocatalysis and photocatalysis. J. Mater. Chem. A 8, 2934–2961. 10.1039/c9ta12776c

[B88] Wei Z. Liu M. L. Zhang Z. J. Yao W. Q. Tan H. W. Zhu Y. F. (2018). Efficient visible-light-driven selective oxygen reduction to hydrogen peroxide by oxygen-enriched graphitic carbon nitride polymers. Energy & Environ. Sci. 11, 2581–2589. 10.1039/c8ee01316k

[B89] Willis D. E. Taheri M. M. Kizilkaya O. Leite T. R. Zhang L. Ofoegbuna T. (2020). Critical coupling of visible light extends hot-electron lifetimes for H_2_O_2_ synthesis. ACS Appl. Mater Interfaces 12, 22778–22788. 10.1021/acsami.0c00825 32338494 PMC7304819

[B90] Wu G. Hu S. Han Z. Liu C. Li Q. (2017). The effect of Ni(i)–N active sites on the photocatalytic H_2_O_2_ production ability over nickel doped graphitic carbon nitride nanofibers. New J. Chem. 41, 15289–15297. 10.1039/c7nj03298f

[B91] Xu J. Chen Z. Zhang H. Lin G. Lin H. Wang X. (2017). Cd_3_(C_3_N_3_S_3_)_2_ coordination polymer/graphene nanoarchitectures for enhanced photocatalytic H_2_O_2_ production under visible light. Sci. Bull. 62, 610–618. 10.1016/j.scib.2017.04.013 36659301

[B92] Yan X. Jia Y. Wang K. Jin Z. Dong C. L. Huang Y. C. (2020). Controllable synthesis of Fe–N_4_ species for acidic oxygen reduction. Carbon Energy 2, 452–460. 10.1002/cey2.47

[B93] Yang H. (2021). A short review on heterojunction photocatalysts: Carrier transfer behavior and photocatalytic mechanisms. Mater. Res. Bull. 142, 111406. 10.1016/j.materresbull.2021.111406

[B94] Yang L. P. Dong G. H. Jacobs D. L. Wang Y. H. Zang L. Wang C. Y. (2017). Two-channel photocatalytic production of H_2_O_2_ over g-C_3_N_4_ nanosheets modified with perylene imides. J. Catal. 352, 274–281. 10.1016/j.jcat.2017.05.010

[B95] Yang S. Verdaguer-Casadevall A. Arnarson L. Silvio L. Colic V. Frydendal R. (2018). Toward the decentralized electrochemical production of H_2_O_2_: A focus on the catalysis. Acs Catal. 8, 4064–4081. 10.1021/acscatal.8b00217

[B96] Ye Y. X. Wen C. Pan J. H. Wang J. W. Tong Y. J. Wei S. B. (2021). Visible-light driven efficient overall H_2_O_2_ production on modified graphitic carbon nitride under ambient conditions. Appl. Catal. B-Environmental 285, 119726. 10.1016/j.apcatb.2020.119726

[B97] Younis S. A. Kwon E. E. Qasim M. Kim K. H. Kim T. Kukkar D. (2020). Metal-organic framework as a photocatalyst: Progress in modulation strategies and environmental/energy applications. Prog. Energy Combust. Sci. 81, 100870. 10.1016/j.pecs.2020.100870

[B98] Zeng X. K. Liu Y. Kang Y. Li Q. Y. Xia Y. Zhu Y. L. (2020). Simultaneously tuning charge separation and oxygen reduction pathway on graphitic carbon nitride by polyethylenimine for boosted photocatalytic hydrogen peroxide production. Acs Catal. 10, 3697–3706. 10.1021/acscatal.9b05247

[B99] Zeng X. K. Wang Z. Y. Wang G. Gengenbach T. R. Mccarthy D. T. Deletic A. (2017). Highly dispersed TiO_2_ nanocrystals and WO_3_ nanorods on reduced graphene oxide: Z-Scheme photocatalysis system for accelerated photocatalytic water disinfection. Appl. Catal. B-Environmental 218, 163–173. 10.1016/j.apcatb.2017.06.055

[B100] Zhang C. Bai J. Ma L. Lv Y. Wang F. Zhang X. (2018). Synthesis of halogen doped graphite carbon nitride nanorods with outstanding photocatalytic H_2_O_2_ production ability via saturated NH_4_X (X = Cl, Br) solution-hydrothermal post-treatment. Diam. Relat. Mater. 87, 215–222. 10.1016/j.diamond.2018.06.013

[B101] Zhang J. Yang H. Gao J. Xi S. Cai W. Zhang J. (2020a). Design of hierarchical, three‐dimensional free‐standing single‐atom electrode for H_2_O_2_ production in acidic media. Carbon Energy 2, 276–282. 10.1002/cey2.33

[B102] Zhang M. M. Lai C. Li B. S. Xu F. H. Huang D. L. Liu S. Y. (2020b). Unravelling the role of dual quantum dots cocatalyst in 0D/2D heterojunction photocatalyst for promoting photocatalytic organic pollutant degradation. Chem. Eng. J. 396, 125343. 10.1016/j.cej.2020.125343

[B103] Zhang W. Liu Z. Chen P. Zhou G. Liu Z. Xu Y. (2021). Preparation of supported perovskite catalyst to purify membrane concentrate of coal chemical wastewater in UV-catalytic wet hydrogen peroxide oxidation system. Int. J. Environ. Res. Public Health 18, 4906. 10.3390/ijerph18094906 34064535 PMC8125239

[B104] Zhao Z. H. Fan J. M. Chang H. H. Asakura Y. Yin S. (2017). Recent progress on mixed-anion type visible-light induced photocatalysts. Sci. China-Technological Sci. 60, 1447–1457. 10.1007/s11431-016-9022-9

[B105] Zheng L. H. Su H. R. Zhang J. Z. Walekar L. S. Molamahmood H. V. Zhou B. X. (2018). Highly selective photocatalytic production of H_2_O_2_ on sulfur and nitrogen for co-doped graphene quantum dots tuned TiO_2_ . Appl. Catal. B-Environmental 239, 475–484. 10.1016/j.apcatb.2018.08.031

[B106] Zinola C. F. Triaca W. E. Arvia A. J. (1995). Kinetics and mechanism of the oxygen electroreduction reaction on faceted platinum-electrodes in trifluoromethanesulfonic acid-solutions. J. Appl. Electrochem., 25, 740–754. 10.1007/Bf00648629

[B107] Zuo G. F. Li B. D. Guo Z. L. Wang L. Yang F. Hou W. S. (2019b). Efficient photocatalytic hydrogen peroxide production over TiO_2_ passivated by SnO_2_ . Catalysts 9, 623. 10.3390/catal9070623

[B108] Zuo G. F. Liu S. S. Wang L. Song H. Zong P. X. Hou W. S. (2019a). Finely dispersed Au nanoparticles on graphitic carbon nitride as highly active photocatalyst for hydrogen peroxide production. Catal. Commun. 123, 69–72. 10.1016/j.catcom.2019.02.011

[B109] Zuo G. Zhang Y. Liu S. Guo Z. Zhao Q. Saianand G. (2020). A beta-cyclodextrin modified graphitic carbon nitride with Au Co-catalyst for efficient photocatalytic hydrogen peroxide production. Nanomater. (Basel) 10, 1969. 10.3390/nano10101969 PMC7600220 33020438

